# Research on hot rolling scheduling problem based on Two-phase Pareto algorithm

**DOI:** 10.1371/journal.pone.0241077

**Published:** 2020-12-28

**Authors:** Wang Chen, Zhang Xiufeng, Zhao Guohua

**Affiliations:** Department of Mechanical Engineering, Hubei University of automotive technology, Shiyan, China; Newcastle University, UNITED KINGDOM

## Abstract

Under the background of excess capacity and energy saving in iron and steel enterprises, the hot rolling batch scheduling problem based on energy saving is a multi-objective and multi constraint optimization problem. In this paper, a hybrid multi-objective prize-collecting vehicle routing problem (Hybrid Price Collect Vehicle Routing Problem, HPCVRP) model is established to ensure minimum energy consumption, meet process rules, and maximize resource utilization. A two-phase Pareto search algorithm (2PPLS) is designed to solve this model. The improved MOEA/D with a penalty based boundary intersection distance (PBI) algorithm (MOEA/D-PBI) is introduced to decompose the HPCVRP in the first phase. In the second phase, the multi-objective ant colony system (MOACS) and Pareto local search (PLS) algorithm is used to generate approximate Pareto-optimal solutions. The final solution is then selected according to the actual demand and preference. In the simulation experiment, the 2PPLS is compared with five other algorithms, which shows the superiority of 2PPLS. Finally, the experiment was carried out on actual slab data from a steel plant in Shanghai. The results show that the model and algorithm can effectively reduce the energy consumption in the process of hot rolling batch scheduling.

## Introduction

Hot rolling is one of the three processes required for iron and steel production. It needs to meet the requirements of multiple objectives and multiple constraints. The hot rolling batch scheduling (HRBS) [1] problem has a significant influence on the quality, cost, and energy consumption of steel production. HRBS is an energy intensive production process in the iron and steel industry. It converts steel slabs at high temperatures in hot rolling process into steel strips. This process consumes a lot of power. Recently, as China has proposed a national policy on energy conservation and emission reduction, and because of rising energy prices, the steel industry must reduce energy consumption and reduce production costs to cope with the above problems during the hot rolling process. The planning task addressed in the HRBS is to generate a production schedule, i.e. to determine the production sequence of the slabs. Hot rolling steel is a customer individual product; the final dimensions of the steel strip after hot rolling are determined by the customer’s requirement. This process requires a different heating temperature; a reasonable planning task of HRBS can effectively solve the problem of energy consumption [2–4].

**Fig 1:**
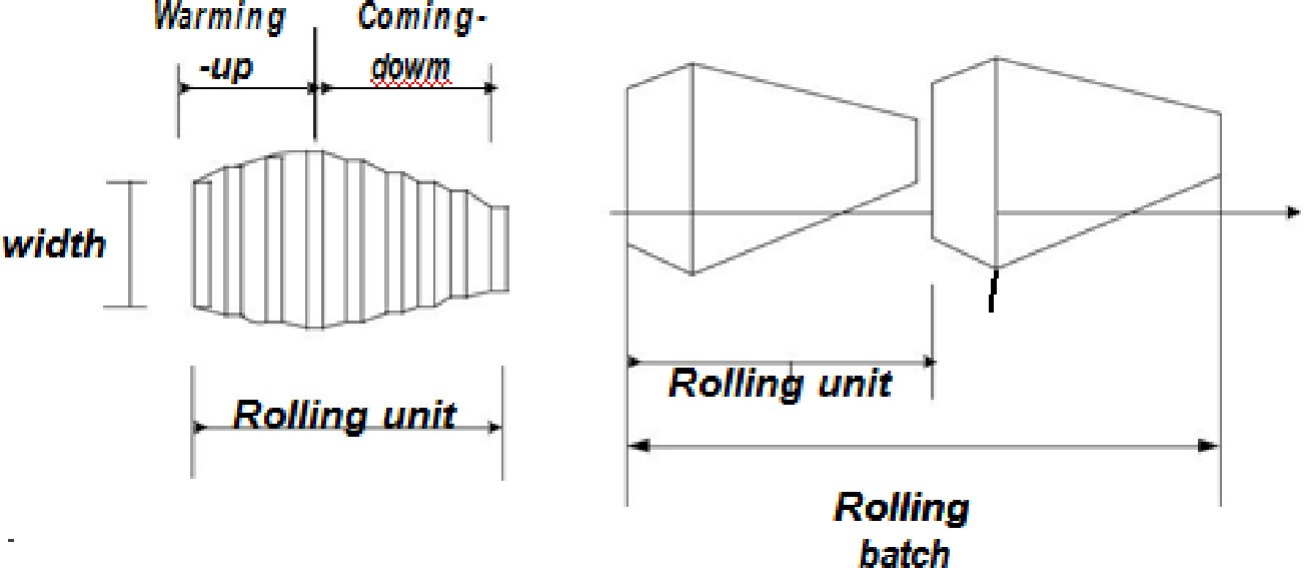
Rolling batch and their structures.

In the HRBS, the order contracts for the original data set and the pre-production contract revenue pool will be grouped into slabs of hot rolling units. The process sequence for a number of rolling units will be then determined according to the constraint conditions of the hot rolling batch scheduling, in order to meet the standards of the continuous casting billet collecting pool (see Fig 1 for the slab structure). The development of the hot rolling batch scheduling program has a significant effect on the product quality, production efficiency, and energy consumption.

**Fig 2:**
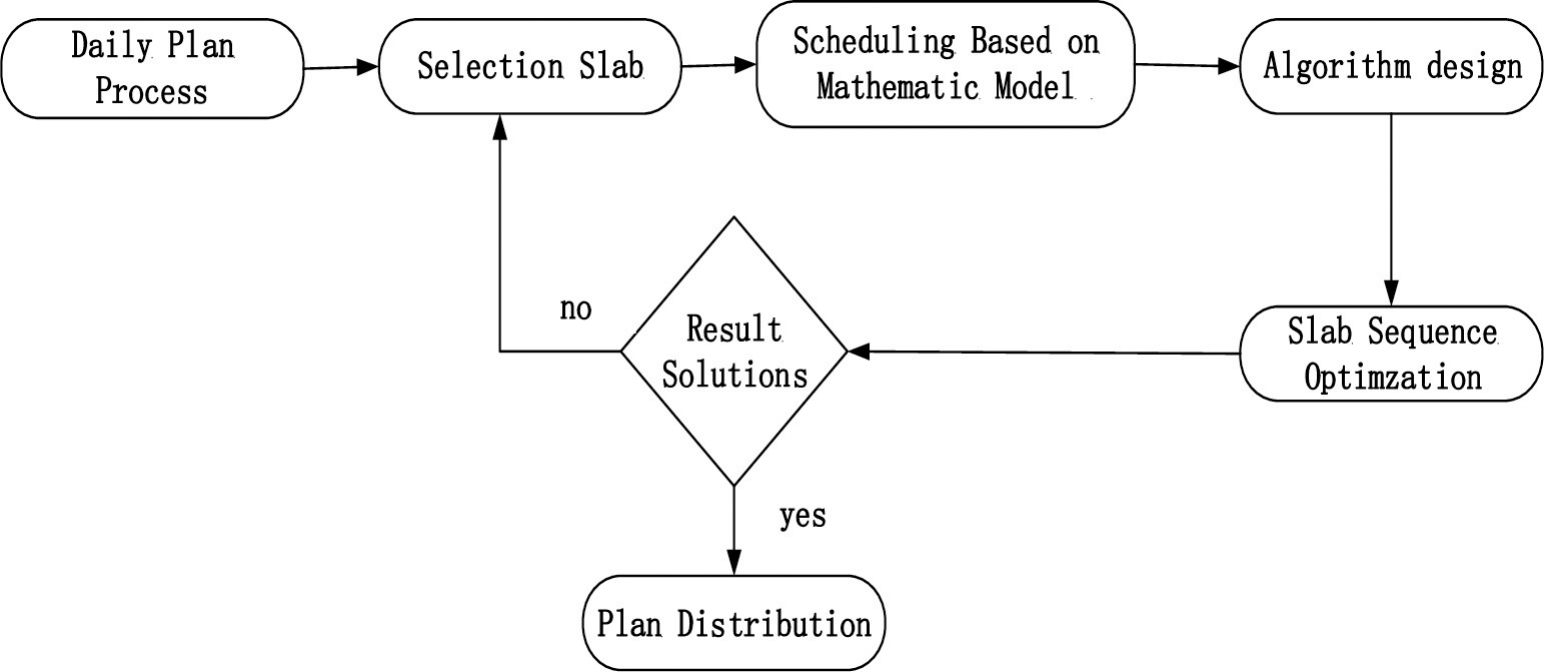
Procedure of hot rolling batch scheduling.

As hot-rolling process is the final procedure among the three steps of steel making, the slab rolling order directly affects the quality of products and energy consumption; therefore, the slab preparation staff should develop a reasonable schedule based on the process, resources, and energy constraints. Since the general delivery time for an order, in an actual enterprise situation is 6–10 days, the plan will not affect the delivery time. Therefore, in this paper, the daily data of the slabs is taken as the object of study, and the slab is divided into several rolling units. In the rolling batch schedule, each rolling unit has strict requirements of slab size, rolling order, steel, convergence of the unit, and rolling volume; and it aims at improving productivity and reducing energy losses, while simultaneously ensuring quality. A reasonable scheduling scheme needs to meet the following objectives:

1. Process conditions to be met: To ensure that the slab meets the surface and physical requirements for the front and rear slab width, thickness, and hardness, for the minimum penalty jump.
2. Resource utilization: To ensure machining quality of the rolling element, maximum roll utilization, reduction in the utilization rate of roll changing, and reduction in the cost of slab production, for the rolling elements of the slab.
3. Saving energy and reducing consumption: In the hot rolling production process, due to the different grades of steel, the hardness and material properties of different slabs, the roll energy-consumption load fluctuation, and the power of the rolling load as the main source of power consumption, the slab rolling sequence will lead to different types of steel rolling at different values power consumption. Therefore, the difference in power consumption between slabs will be the minimum penalty jump. The transition between the slabs for power consumption should be smooth, and should not have any repeated jumping. The process for hot rolling batch scheduling preparation is shown in Fig 2.

The HRBS will greatly affect the hot rolling energy consumption issues. Also, due to the relatively few studies on the energy consumption optimization of the HRBS, motivation of this research prompted us to further study the energy consumption of HRBS based on the new optimization model. The model contains the optimization objectives and constraints of process, resources and energy consumption, multi-objective optimization algorithm was designed and solves the model by this paper.

The main contributions of this paper are as follows:

1. Considering the previous research, the minimum number of rolling plans for minimum power loss and the minimum processing per ton of steel plate, HRBS is summed into a hybrid prize collecting model.
2. We used the 2PPLS algorithm to solve the constructed HPCVRP model. The effectiveness of the proposed algorithm was verified by comparing it with the other MOEAs. In this study, we used the MOEA/D-PBI algorithm [12] to decompose the HPCVRP, and to enhance the efficiency of finding the local optimal solution, we then used the MOACS and Pareto local search (PLS) method to obtain the final solution.

Finally, the simulation data was compared with the manual scheduling data (actual data of an iron and steel enterprise in Shanghai) to ensure further optimization of the hot rolling batch scheduling problem.

The remainder of this paper is organized as follows. The literature review and related work is presented in Section II. The mathematical model for the hot rolling batch scheduling is presented in Section III. Section IV presents the 2PPLS algorithm. Section V includes the experimental studies for demonstrating the efficiency of the proposed method in addition to some discussion. Finally, the conclusions are presented in Section VI.

### Literature review and related work

This paper reviews the literature from two aspects of hot rolling scheduling model and solving algorithm.

In hot rolling scheduling modeling, HRBS is a typical non-deterministic polynomial (NP-hard) combinatorial optimization problem with a large number of multi-objectives and constraints (Lopez, Carter & Gendreau, 1998), it is almost impossible to produce optimal scheduling solutions by manual scheduling or intelligent optimization methods [5]. Atilla Özgüra et al reviewed 90 hot rolling mill scheduling articles from 1989-2020.Diversity in optimization methods, constraints incorporated in the analysis and level of abstraction and data availability are analyzed [6]. Quan Ke Pan, Liang Gao and Wang Ling addressed a hot-rolling scheduling problem from compact strip production pro- cesses. A mathematical model that consists of two coupled sub-problems is presented, a multi-objective evolutionary algorithm (MOEA/D) is developed to find Pareto optimal or near–optimal solutions of the sheet-strip sequencing problem [7]. A new mixed-integer problem formulation model for hot rolling mill that focuses on energy consumption was established by Karen Puttkammer et al. (2016). They used the greedy random adaptive search algorithm to solve the model, and achieved good results with respect to energy consumption [8]. From the above analysis, it can be seen that the HRBS is more and more closely related to energy consumption. HRBS is mostly approximate mathematical model.

In terms of solving algorithm, Liu et al (2015) used a combination of particle swarm optimization (PSO) and simulated annealing algorithm (SA) to solve the HRBS. The Metropolis criterion was introduced to search for Pbest (Pbestis the local optimization solution) and Gbest (Gbestis the global optimization solution) twice. The algorithm obtained good results, when compared to the results of manual scheduling. However, the solution process uses a simple weighting method, in which the weight coefficient is given by experience, to convert the multi-objective problem into a single objective problem without considering the consistency between multiple objectives, to obtain the scheduling results; therefore, it needs to be optimized further [9]. Zangari M, Pozo A, Santana R, et al (2017) have presented a preliminary study on an algorithm based on MOEA/D framework and the bio-inspired metaheuristic called binary ant colony optimization (BACO) [10], The results have shown that the proposed MOEA/D-BACO has outperformed MOEA/D, which uses genetic operators, in most of the test instances. As the HRBS is an NP-hard combinatorial optimization problem, and has multi-objectives and multi-constraints; the ACO is one of the best algorithms to solve the classic TSP problem. Rui Zhang et al (2020) proposed a hybrid metaheuristic algorithm, which combines ant colony system and enhanced local search to provide an efficient solution to the HRBS [11]. Tan et al (2019) have presented a hybrid algorithm that combines mixed integer programming and constraint programming is designed to solve each subprolem [12]. The hybrid algorithm and enhanced local search strategy can be used to solve the HRBS.

As discussed above, the traditional hot rolling process consumes a large amount of energy and cannot meet the requirements of energy saving and consumption reduction. We must analyze and study these characteristics to build a new mathematical model. In addition, we require a new algorithm to solve HRBS when the number of objectives is more than 3 and contains many constraints. In this paper, the problem of hot rolling batch optimization is formulated as a hybrid multi-objective prize collecting vehicle routing problem (HPCVRP). We propose a two-phase Pareto local search (2PPLS) algorithm for the real-world multi-objectives optimization problem. In the first phase, the algorithm generates a set of high-quality solutions by MOEA/D-PBI, and in the second phase, we employ the Pareto local search (PLS) method to generate approximate Pareto-optimal solutions

### Problem modeling

At present, there are many models for VRP research, but the considerations are often rather single and far from wide practical application. However, there are many factors to be considered in HRBS.

1. To fully ensure the capacity of rolling unit, it is necessary to set a vehicle path model with unknown number of vehicles;
2. To fully reflect the preferential processing temperature of the slab, bonuses collection mode, that bonus to collect vehicle routing model;
3. To meet the wear life constraints of the roll, a vehicle path model that can constrain the vehicle path, that is, the ability to restrain needs to be adopted.

**Fig 3:**
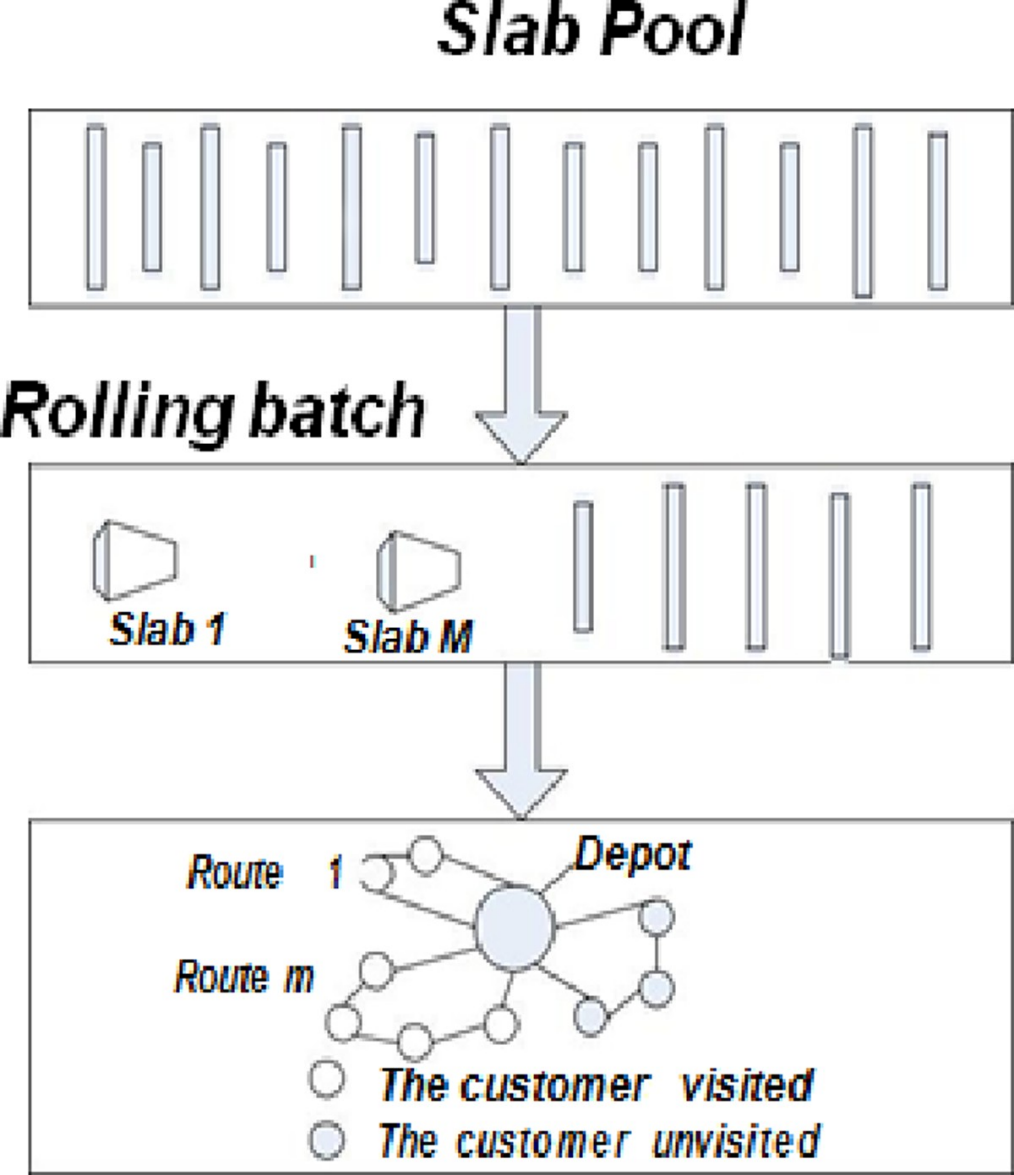
HPCVRP and HRBS.

In this paper, a new hot rolling optimization model is established according to the characteristics of hot rolling batch scheduling. The HPCVRP model is combined with the characteristics of VRP and PCTSP. Each slab corresponds to a store, each rolling unit corresponds to a car, and each store requires a corresponding slab rolling length, which is the distance between the penalty values of the corresponding store slabs. As shown in Fig 3, the distribution centre of the distribution vehicles must pass and eventually be returned to the distribution centre in HPCVRP. Thus, it is a closed loop process; however, since the hot rolling process is an open loop process, the “virtual slab” concept is introduced at the beginning and end of the slab rolling process. Each slab must be and can only be placed in a rolling unit. In order to describe the mathematical model, the parameters are defined as follows:

Decision variables: x_ijk_—in the rolling units k, the slab i is followed by j; y_ik_—the rolling units k contains the slab i;

HRBS can be formulated as the following multi-objective HPCVRP model:

$$S_{1}=\min\sum_{k=1}^{M}\sum_{i=0}^{n}\sum_{j=0}^{n}d_{ij}.x_{ijk}$$ (1)

$$S_{2}=\min\sum_{i=1}^{n}p_{i}\left(1-\sum_{k=1}^{M}y_{ik}\right)$$ (2)

$$S_{3}=\min\sum_{k=1}^{M}\sum_{i=0}^{n}\sum_{j=0}^{n}P_{ij}^{e}x_{ijk}$$ (3)

$$\sum_{i\in N}x_{ijk}=y_{ik},j\in N,k\in M$$ (4)

$$\sum_{j\in N}x_{ijk}=y_{ik},i\in N,k\in M$$ (5)

$$\sum_{i=1}^{n_{k}}\sum_{j=1}^{n_{k}}x_{ijk}\leq n_{k}-1$$ (6)

$$y_{0k}=1,k\in M$$ (7)

$$\{\begin{array}{l} \sum_{i\in N}y_{ik}Q_{i}\geq QS,k\in M\\ \sum_{i\in N}y_{ik}Q_{i}\geq QL,k\in M \end{array}$$ (8)

$$\left\{ \begin{array}{l} x_{ijk}\in \left\{0,1\right\}i,j\in N,k\in M\\ y_{ik}\in \left\{0,1\right\}i\in N,k\in M \end{array}\right.$$ (9)

Objective (1) is to minimize the total penalties caused by the jumps in width, gauge, and hardness between adjacent slabs. Objective (2) is to minimize the penalties due to unscheduled slabs. Objective (3) is to minimize the total penalties caused by the jumps in the power consumption (per ton of steel) slabs. Constraints (4)–(5) specify the sequence of a rolling unit. Constraints (6)–(7) ensure that the slabs are scheduled only once, while the virtual slab 0 must be scheduled in each rolling unit. Constraint (8) indicates the capacity constraint of each rolling unit. Constraint (9) ensures that the decision variables x_ijk_ and y_ik_ take the integer values of only 0 or 1.

## Solution algorithm

In the HPCVRP, the continuous slab and rolling unit are considered as the service customers and delivery vehicles, respectively. During the rolling process, the total value of the width, thickness, and hardness of the adjacent slab represents the transportation cost. To ensure minimization of the energy cost of production, the hot rolling slab not only needs to meet the process and resource conditions, but also needs to ensure maximum energy saving. It can be seen from equation (1)-(9) that the HPCVRP model contains three objectives and multiple constraints, and the optimization of this model is many objective optimization problems (MaOPs). As the common intelligent algorithms, such as the ant colony algorithm, genetic algorithm, etc., have poor performance in dealing with MaOPs, we need to use a new algorithm to improve the performance of combination optimization problems, and to deal with MaOPs.

### The proposed Two-phase Pareto search algorithm

#### Two-phase Pareto search algorithm

**Fig 4:**
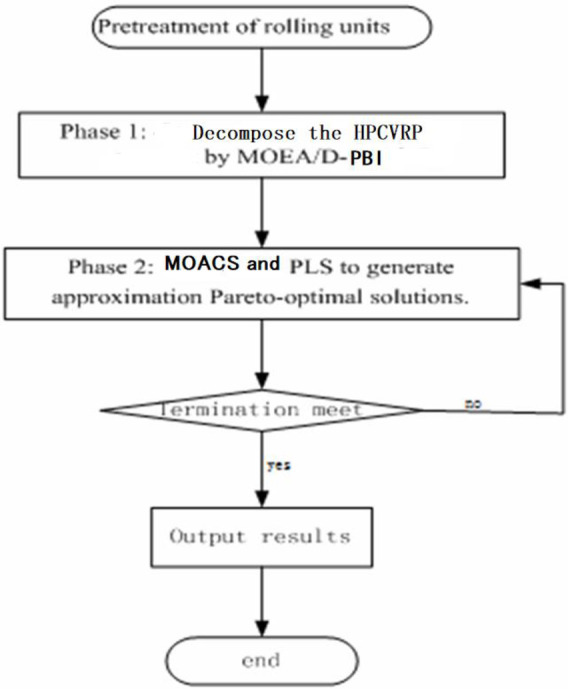
Procedure for Two-phase Pareto algorithm.

HPCVRP is a combinatorial optimization problem, when the number of slabs is large, it will bring "combinatorial explosion", and there are more optimization objectives problem, In addition, for the MaOPs, the weighting method is generally used, but the dimensions of the multi-objective in the rolling plan model are not the same, and the weight coefficient of the objective is difficult to be determined reasonably. So it is very difficult to solve it accurately. In this paper, we present 2PPLS to find a suitable approximation solution to the above problem. The flow chart for the algorithm is shown in Fig 4. The 2PPLS are as follows:

1. Phase 1: Decompose HPCVRP by MOEA/D-PBI.
2. Phase 2: MOACS and PLS to generate the approximate Pareto-optimal solutions.

#### First phase

Ant colony optimization has been more and more used in MOPs. An evolutionary algorithm using ant colony by decomposition (MOEA/D-ACO, Ke et al. 2013) [11] for MOPs was proposed. Recently, An MOEA/D-ACO with PBI for MaOPs was proposed by (Ling and Wang.2018). As the number of objectives in HPCVRP is more than three, the MOEA/D-ACO uses the weighted sum and Tchebycheff approaches to solve the problem, but the correlation between the algebraic and the geometrical interpretation of the weight sets is not valid. In this paper, we introduce the PBI approach to MOEA/D. Therefore, we decompose HPCVRP into multiple sub-problems through MOEA/D-PBI algorithm. The purpose of PBI is to find the intersection of the top boundary and a group of lines. When these lines are uniformly distributed, the interval of the result provides an approximate solution for the Pareto optimal boundary of the whole population. This is helpful to deal with the non-convex problem of Pareto optimal bound. The optimization problem of the PBI approach is defined as:

$${\text{Minimize}\;\text{g}}_{\text{pbi}}\left(\text{x}|\text{w,z}*\right)={\text{d}}_{1}+{\theta \text{d}}_{2}$$ (10)

Subjecttox∈Ω

Where

d1=∥(F(x)−z*)Tw∥/∥w∥

**Fig 5:**
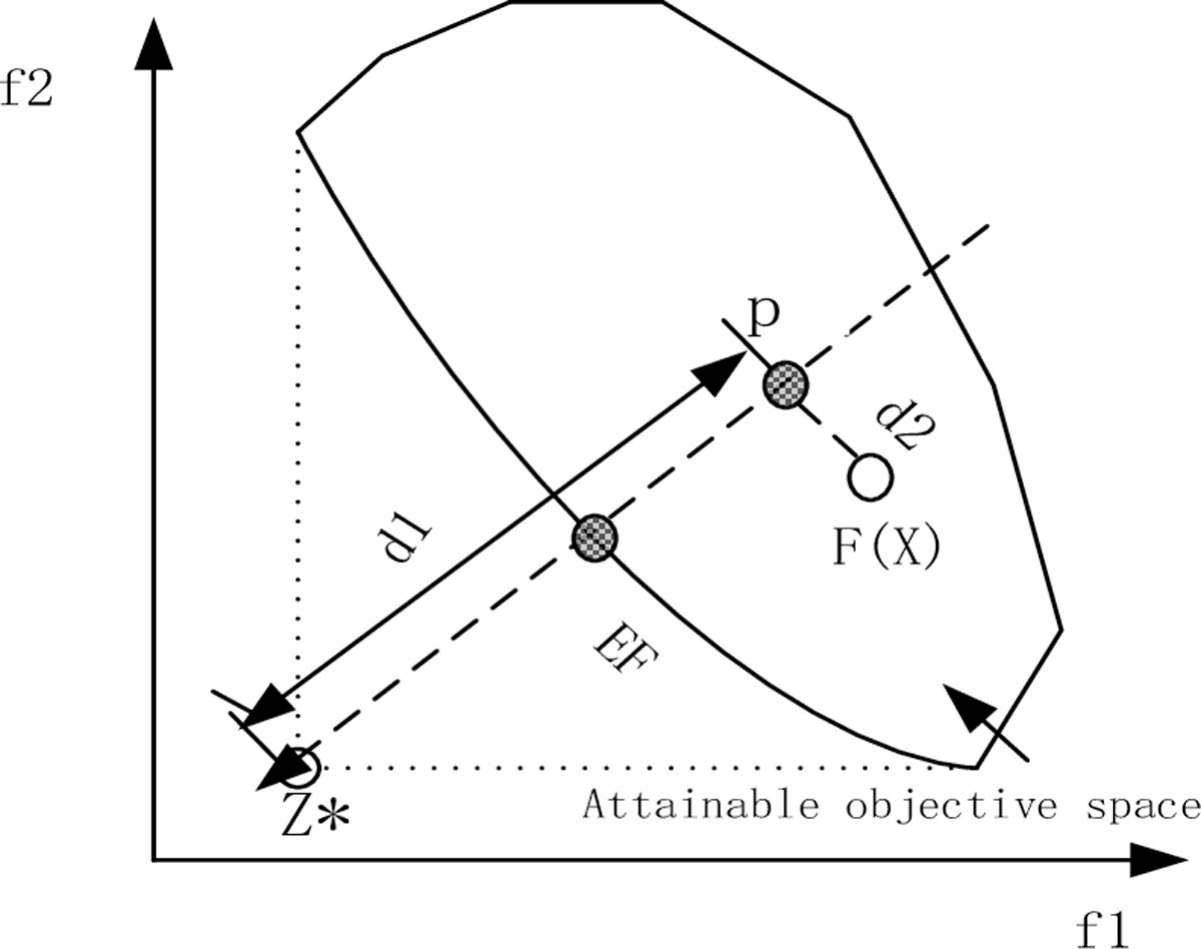
Illustration of PBI approach [13].

d2=∥F(x)−(z*+d1w/∥w∥∥

z* = (z_1_ …z*_m_)^T^ is the ideal objective vector with z_i_*<minf_i_(x), x∈ Ω, i∈ {1 …m}, Fig 5 illustrate d_1_ and d_2_ of a solution x_i_. G_pbi_(x|w,z*) is a composite measure of x for both convergence and diversity, d_1_ is used to measure the convergence of x_i_ towards the EF, d_2_ is a kind of measure for the population diversity. The distance between d_1_ and d_2_ is controlled by the parameter θ = 5.0 (θ is a penalty parameter).

MOEA/D-PBI was shown in algorithm1.

### Algorithm 1 MOEA/D-PBI

(1) Initialization

(2)Decomposition by PBI approach

(3)Solution construction

(4)Update of EP

(5)Update of pheromone

(6)Check the solutions in the neighbourhood and update the solutions

(7)Termination: judgment and output EP(a set of high-quality solutions X_S_)

#### Second phase

MOACS is used to solve the HPCVRP. Each ant is responsible for solving one sub-problem. During the search, each ant also records the best solution found so far for its sub-problem. An ant updates its current solution if it has found a better one in terms of its own objective. The path (rolling plan) constructed by ant i. The PLS method is a local search algorithm developed by various authors [14]. In PLS, the neighbourhood of every solution is explored. If the neighbour is not dominated by a Pareto-optimal solution, then the neighbour is added to the population and the set of approximate Pareto-optimal solutions. The algorithm stops when it is no longer possible to find any more dominated neighbours from the population. The PLS does not prescribe any numerical parameters based on the notion of Pareto local optimum set [15–17]. In this paper, the set of Pareto-optimal solution denoted by X_E_ contains all the approximate Pareto-optimal solutions. The main purpose for the second phase is the generation of the approximate Pareto-optimal solutions X_E_. The pseudo-code of the PLS is shown in algorithm 2.

### Algorithm 2 Pareto Local search algorithm

Input: initial population X_S_

Output: an approximate Pareto-optimal solution X_E_

(1) X_E_←P_ini_ //initialization of X_E_ and population P_0_ with the initial population P_ini_

(2)P_0_←P_ini_

(3)P_a_←∅//initialization of an auxiliary population P_n_

(4)added←true //added is a Boolean variable

(5)**while** P_0_≠∅**do**// generation of all the neighbours p_n_ of each solution *p*_*0*_∈P_0_

(6)**for all** *p*_*0*_∈P_0_**do**

(7)**for all** p_n_∈N(*p*_*0*_) **do**

(8) **if** z(*p*_*0*_)>z(p_n_) **then**

(9) **for** all x∈X_E_ **do** // add solution step 9–step16

(10) **if** z(x)≤z(*p*_*0*_) **then**// a solution x, its evolution z(x)

(11)added←false

(12) Break

(13)**if** z(*p*_*0*_) ≺ z(x) **then**

(14) X_E_←X_E_\ {x }

(15) **if** added = true **then**

(16) X_E_←X_E_∪{*p*_*0*_}

(17) **if** added←true

(18) **if** z(x) ≤z(*p*_*0*_) add solution step 18–24

(19)  added←false

(20)  break

(21) **if** z(*p*_*0*_) ≤z© **then**

(22) X_E_←X_E_\{x}

(23)**if** added = true **then**

(24) X_E_←X_E_∪ {*p*_*0*_}

(25) P_a_←∅// re-initialization of P_a_

#### Solving process

According to the 2ppls algorithm mentioned above, it is used to solve the HPCVRP model. The specific process is shown in algorithm 3.

### Algorithm 3 2PPLS for HPCVRP

Input: initial parameters

(1) Decomposing HPCVRP into several sub-problems by MOEA/D-PBI

(2)Select ant i from X_E_ and starts from virtual slab 0 and selects the slab with the largest width from the candidate slab

set as the first slab of the k-th rolling unit main material

(3) Ant i select the next slab from the feasible slab set NF according to the state transfer strategy

If N_F_ = 0, then it returns to virtual slab 0 and moves to the next step. Otherwise, continue to select the next slab

(4) Let t = t + 1, if t ≤ m, turn to step (2) to start the construction of the next rolling unit.

After all ants complete the path construction, if t > m, according to the Pareto local search strategy,

the path (rolling plan) constructed by ant i is searched locally to improve the quality of the solution

(5) Update the pheromone of the non dominated solution successfully updated to the external file in each iteration.

If the algorithm meets the termination conditions, output the non dominated solution and the corresponding approximate Pareto front end in the external file and stop the algorithm; otherwise, transfer to step 2

## Simulation results

In this section, we compare the 2PPLS algorithm with five state-of-the-art algorithms, including MOEA/D-ACO^W^ (Ke et al. 2013), MOEA/D-ACO^T^ (Ke et al. 2013), IABC (An improved artificial bee colony algorithm,Li et al.,2016), HVNS (A hybrid variable neighbourhood search algorithm, Zhang Biao et al 2018), and HPSA (Liu et al. 2016). There five algorithms are solutions to HRBS more classic algorithms [18–20]. The test problems are introduced in Sect. 5.1. Sect 5.2 describes the quality indicators. The five state-of-the-arts algorithms used for comparison and the corresponding parameter settings are briefly introduced in Sect. 5.3.

### Parameter settings

The parameters for the six MOEAs considered in this study are listed below.

1. Parameter settings in MOEA/D-PBI: set T = 0.3, α = 1, β = 10, ρ = 0.95, r = 0.9, and θ = 5.0.
2. A few parameter settings in MOEA/D-ACO^W^ and MOEA/D-ACO^T^: T = 0.3, α = 1, β = 10, ρ = 0.95, and r = 0.9. The other parameters can be obtained from the reference (ke et al. 2013).
3. Some common parameter settings for IABC: Population = 50, Number of neigh boring solutions = 3, local search times for scout bees = 10 (Li et al. 2016).
4. HVNS: the number of neighbourhood structures = 4, the number of neigh boring solutions generated in the FFO
  (Fruit fly optimization algorithm)-based local search (d) = 5, the number of iterations for conducting the FFO-based local search in the given neighbourhood © = 20, the number of consecutive iterations of failing to update the incumbent solution (L) = 50 (ZhangB et al, 2018).
5. Parameter settings in HPSA: λ = 0.95, c1 = c2 = 1.5, T_ini_ = 1000, as suggested in (Liu et al. 2016).
6. Number of runs and termination condition: The algorithms were programmed in *PlatEMO* (ye Tian et al 2016), and each algorithm was independently run 30 times for each test instance and stopped after 300 generations. The total number of iterations of the 2ppls algorithm is 30 times in the first phase (MOEAD/IACO) and the second phase (Pareto local search) respectively. All the algorithms were executed on a desktop PC with a 2.6-GHz CPU, 8-GB RAM, and Windows 10.
7. Number of points in Monte Carlo sampling: It was set to 1,000,000 to ensure precision [21–23].

#### Results and discussion

**Fig 6:**
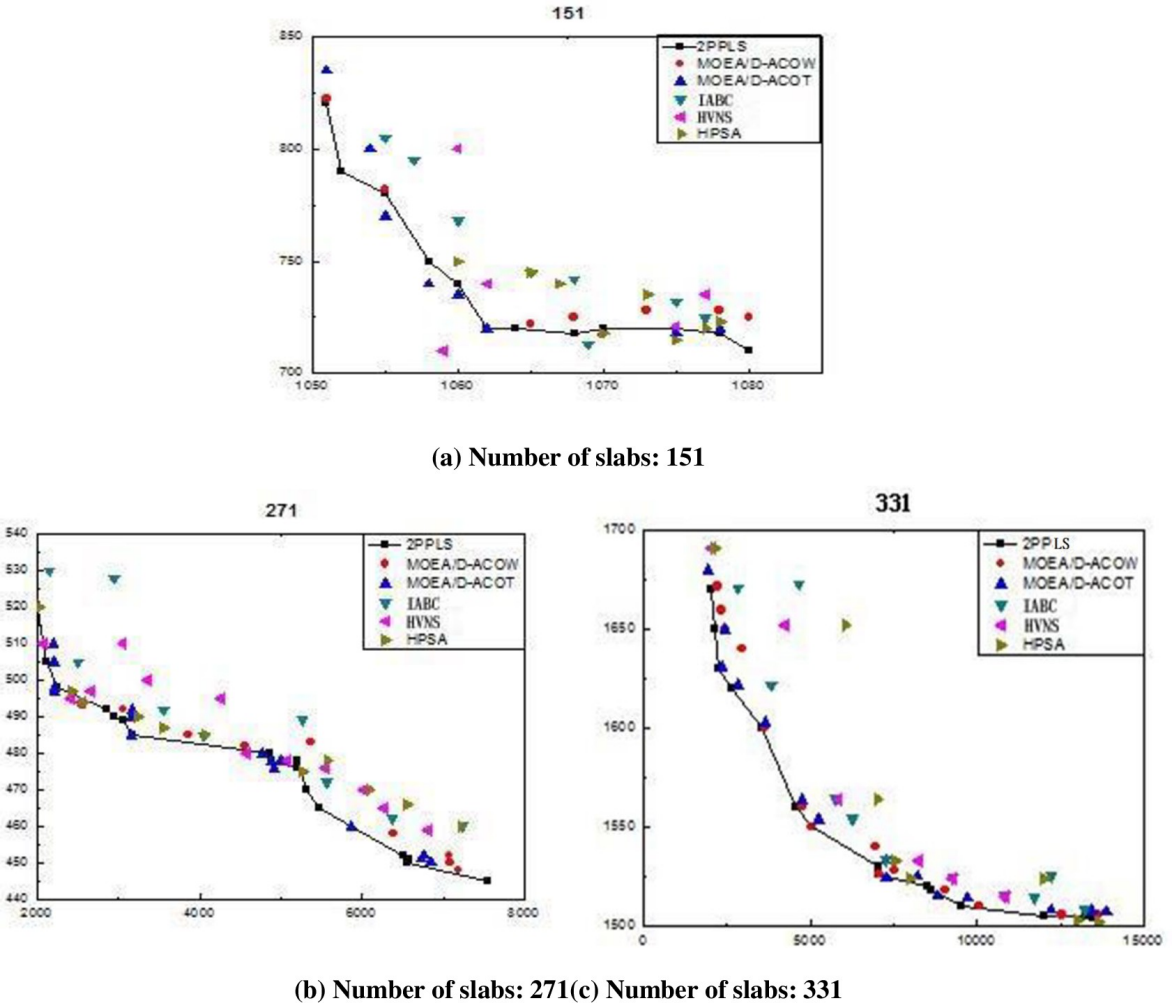
Approximate Pareto-optimal fronts returned by the six algorithms.

To prove the advantages of the 2PPLS algorithm in solving HRBS, three groups of practical production data were collected from Shanghai Baoshan Iron & Steel Co., Ltd. The data consisted of different number of slabs of 151, 271, and 331. They were divided into three problems. The problems were named based on the number of slabs. E.g., Problem 331 contains 331 slabs. The approximate front returned by each algorithm corresponding to each batch-scheduling problem is shown in Fig 6, in which the horizontal axis denotes the total penalties caused by the jumps between adjacent slabs, and the vertical axis denotes the penalties for unscheduled slabs, the conclusions are given below.

1. 2PPLS has the best convergence performance, as its approximate fronts cover most of those returned by the other algorithms, especially for the larger scheduling problems. Furthermore, 2PPLS can generate some non-dominated solutions with the collection of higher prizes, which is important for HPCVRB, because collection of higher prizes imply that more prior slabs can be scheduled in the rolling batch.
2. MOEA/D-ACO^W^ can also generate good solutions for the smaller scheduling problems such as 151; however, it has a worse convergence performance for the larger scheduling problems such as 331.
3. MOEA/D-ACO^T^ can obtain well-spread approximate fronts; however, some of its solutions were dominated by 2PPLS.
4. IABC, HVNS, and HPSA are good if and only if the number of slabs is small, and only a few solutions are better than that of 2PPLS.
5. It can be seen from Fig 6 that 2PPLS dominates in most solutions and that the advantage of 2PPLS is more pronounced as the number of slabs increases.

#### Actual data validation

Table 1 shows the results between, 2PPL, MOEA/D-ACO^w^, HPSA and manual scheduling method. Table 2 shows the results of a comparison between the 2PPLS, the manual algorithm for HRBS, the results of the manual and 2PPLS scheduling methods. From these results, it can be seen that the solutions obtained from the 2PPLS are better than those from the other algorithms. Compared with manual scheduling algorithm, it can be noticed that the values of objective1, objective2, and objective3 were reduced by 15.43%, 15.98% and 10.01%, respectively for the unit 151, by 19.75%, 21.2%, and 15.89%, respectively for the unit 271, and by 23.98%, 23.33%, and 17.28%, respectively for the unit 331. The energy saving rates was 10.01%, 15.89%, and 17.28%. These results and comparison show that 2PPLS is definitely better than the manual algorithm, in terms of convergence precision, when searching for optimal results of HPCVRP.

**Table 1:** Comparison results between2PPL, MOEA/D-ACO^w^, HPSA and Manual scheduling method.

	Units	Objective 1 value	objective2 value	objective3 value	cup time
2PPLS	151	1780	820	650	30s
	271	2105	671	540	
	331	5994	923	881	
ACO^w^	151	1530	732	41	75s
	271	1923	545	432	
	331	4875	852	780	
HPSA	151	1580	793	630	55s
	271	1978	642	510	
	331	5775	897	820	
Manual	151	2105	976	723	1200s
	271	2623	852	642	
	331	7885	1204	1065	

**Table 2:** Slab sequence obtained from manual and 2PPLS.

Manual	6	7	8	9	10	14	19	18	16	17	27	21	26	22	23
2PPLS	6	7	8	9	10	14	21	26	22	23	25	20	24	5	4
Manual	25	20	24	5	4	1	2	3	32	33	34	31	35	11	13
2PPLS	1	2	3	32	33	34	31	35	18	19	11	12	13	15	16
Manual	15	12	29	28	30										
2PPLS	17	27	28	29	30										

## Conclusions

The major contribution of this paper is proposal of an HPCVRP module for HRBS. With regard to saving energy, reducing consumption, and improving production efficiency, the HPCVRP model can reduce the energy consumption in the process for hot rolling batch scheduling, while satisfying the process requirements and resource utilization rate. A set of high-quality solutions is generated by MOEA/D-PBI in the first phase of the 2PPLS. Due to the number of model optimization objectives, the PBI aggregate function was selected to decompose the objectives. In the second phase, we employ the MOACS and PLS method to generate approximate Pareto-optimal solutions. Based on the actual slab data for scheduling, it could be seen that the results of the 2PPLS scheduling method are significantly better than those of the manual scheduling method. Therefore, the planner can select a satisfactory solution based on their experience or preference. The results show that the 2PPLS is better than the manual method with regard to scheduling performance. Our future work aims at analysing further, how to deal with HPCVRP and dynamic constraint processing, and to optimize the number of objectives more effectively.

## Abbreviations

HPCVRP: Hybrid Price Collect Vehicle Routing Problem
2PPLS: Two-phase Pareto search algorithm
MOPs: Multi-objective optimization problems
PLS: Pareto local search
MOEA/D: PBI, PBI with MOEA/D algorithm
PBI: Penalty-based boundary intersection
J: The number of groups
m_0_: the number of objectives
X_E_: Approximate Pareto-optimal solutions
EP: External population
X_S_: Population from EP
p_ini_: Select population from X_S_
p_n_: Neighbour’s p_ini_
P_a_: Auxiliary population
θ: Penalty parameter
m: The number of rolling units
N: The set of all candidate slabs
N_F_: Feasible slab assembly
M: the set of rolling units
n: The number of rolling units slab
g_c_(x_t_): The c-th inequality constraint
h_k_ (x_t_): The k-th equality constraint
p_ine_: The number of inequality constraints
q_equ_: The number of equality constraints
N: The set of all candidate slabs, N = (i = 0, 1, 2…n), where n is the slab quantity and i = 0 is a virtual slab
M: The set of rolling units, M = {0, 1, 2… m}, where m is the number of rolling units.
K_r_: rolling unit, (k = 1, 2, 3…m)
d_ij_: The penalties to the roll slab j immediately after slab i, where d_ij_ = P^w^_ij_+P^g^_ij_+P^h^_ij_, P^w^_ij_、P^g^_ij_、P^h^_ij_ represent the penalties caused by the jumps in width, gauge, and hardness when moving from slab i to slab j
P^e^_ij_: The penalties caused by the power consumption (per ton of steel) when moving from slab i to slab j
P_i_: The prize (penalty) obtained if slab i is (is not) scheduled in the rolling batch. P_i_ represents the desirability of the processing slab i, and can be set as a product of the priority and the rolling length of slab i. Therefore, a slab with a higher priority and a longer rolling length obtains a higher price (penalty) than that with a lower priority and a shorter rolling length.
Q_i_: The rolling length of slab i, where i∈ N
Q_L_: The lower bound of the rolling length of the slabs scheduled in each rolling unit
Q_S_: The upper bound of the rolling length of the slabs scheduled in each rolling unit
